# Fitting a Davis or Logan Crown

**Published:** 1903-04-15

**Authors:** A. F. Miller


### DENTAL REGISTER.

Vol. LVII. April 15, 1903. No. 4.

### CLINICS AT THE OHIO STATE MEETING.

### FITTING A DAVIS OR LOGAN CROWN.

BY DR. A. F. MILLER.

Dress the stump of the root as desired, and select a Davis crown to be used. Grind the crown to fit the end of the root. Now take an accurate wire measurement of the end of the root and preserve its form when removing it. Place this wire form on the end of a piece of hard hickory wood and with a hammer bury it into the flat surface of the end of the stick. This will leave an imprint of the wire, giving exact size and form of root. Cut away the wood until you have a duplicate of the root end in the carved wood stick. Ream out the canals to desired form and size so that a suitable post may be inserted. With a pair of small compasses locate the canal in the root and transfer this measurement to the wood block, and with suitable burs reproduce the location, form and size of the canal in the wood block. Now, select a suitable pin and conform it to the root-canals and adjust the porcelain crown over it to a fit. Next fill the cavity of the porcelain crown with cement and place it on the pin which is left in the wooden mimic of the tooth. When the cement has set remove the post and crown together from the block and grind its sides until they conform to the exact shape of the natural root. The crown is then to be set in the usual way as a pin crown which has had its post baked into the porcelain with the exception that it has been already ground to an accurate fit. We would suggest that the same result might be had by securing a fusible alloy counterpart of the root stump and the grinding the crown to this.—Ed.

